# Supplementary material for: Liver function indicators in patients with breast cancer before and after detection of hepatic metastases-a retrospective study
Source: PLoS One. 2023 Mar 3;18(3):e0278454. doi: 10.1371/journal.pone.0278454 (PMC9983906; doi:10.1371/journal.pone.0278454)
Supplement: S1 File — (DOCX) [file pone.0278454.s001.docx]

| n Mean (Std) Median | 6 months prior to BCLM diagnosis | At the time of BCLM diagnosis | 12 months after BCLM diagnosis |
| --- | --- | --- | --- |
| AST U/L | 68 34.2 (22.3) 26 | 104 65.4 (72.6) 35.5 | 74 77.7 (116.9) 34.5 |
| ALT U/L | 69 32.8 (33.3) 22 | 103 47.4 (47.6) 30 | 73 41.3 (40.1) 28 |
| GGT U/L | 69 70.3 (91.8) 36 | 103 251.9 (486.3) 53 | 74 232.7 (320.4) 83 |
| LDH U/L | 58 261.2 (235.1) 198 | 91 398.0 (327.5) 266 | 60 529.5 (1009.1) 237.5 |
| AP U/L | 68 111.9 (66.8) 93 | 103 193.7 (207.5) 113 | 71 217.4 (262.9) 116 |
| Albumin g/L | 48 39.2 (4.8) 39.6 | 89 38.7 (5.5) 39.6 | 62 35.8 (7.2) 36.45 |
| Bilirubin mg/dL | 68 0.6 (0.4) 0.47 | 101 0.9 (1.8) 0.49 | 73 1.6 (3.3) 0.61 |
