# Supplementary material for: Liver function indicators in patients with breast cancer before and after detection of hepatic metastases-a retrospective study
Source: PLoS One. 2023 Mar 3;18(3):e0278454. doi: 10.1371/journal.pone.0278454 (PMC9983906; doi:10.1371/journal.pone.0278454)
Supplement: Attachment — Submitted filename: Leser et al 2022.docx [file pone.0278454.s002.docx]

In the manuscript titled “Liver function indicators in patients with breast cancer before and after detection of hepatic metastases”, Leser et al. have reported a clinically relevant prognostic significance of liver functions in breast cancer liver metastasis. A retrospective temporal analysis of different liver enzymes Aspartate aminotransferase, alanine aminotransferase, gamma-glutamyltransferase, lactate dehydrogenase and alkaline phosphatase, and albumin protein are important parameters to predict liver metastasis in breast cancer patients. However, it is expected that the authors generate more figures from the data compiled in the tabular form for a better understanding of the results. In addition, the correlation plot must be shown if both aminotransferase and albumin correlate with the patient’s survival. Irrespective of significance, both univariate and multivariate analysis must be presented to find the significance and correlation of proposed prognostic liver function proteins/enzymes. Unfortunately, the current form of the manuscript is not acceptable. However, the following are comments that must be addressed so that the manuscript can be reconsidered for publication in PLOS One. Please see the following comments:

1. Add continuous line numbers in the manuscript so that comments can be addressed to the page and line number.

2. References cited do not cover the recent publications in the field. For example, references for statistical values presented in the introduction are old, including refs.1-4. Please include the most up-to-date information with recent citations.

3. It is not clear what the treatment was for the patients diagnosed with liver metastasis for 12 months post-diagnosis. Is there information available on whether these patients were considered for surgery or any other treatment modality?

4. Description of each enzyme and protein has been given in the results. However, authors are suggested to plot the values to make them clearer and more understandable.

5. Baseline value for each parameter in normal individuals can be merged in table 2, and data can be plotted in the form of bar graphs.

6. Figure legends are missing for figures given in the manuscript. Please provide the figure legends, including the statistical significance.

7. Font size and type are not consistent in eh manuscript and figures. Authors are suggested to revise the manuscript with the same font type and size as per the journal’s guidelines.

8. It has not been mentioned what the label on the Y-axis of figure 2 is. Please add the Y-axis details.

9. Discussion needs to be elaborated with overall more citations of recent work in the field.

10. Authors are suggested to explain why enzyme and protein levels went down after 12 months of diagnosis of BCLM, trending towards the values observed 6 months prior to the diagnosis of BCLM. Is this an effect of treatment in these patients? Were there patients where progressive liver metastases showed a positive correlation with elevated enzyme and protein levels?

11. Page 8, Line 8, the word prediction should be replaced by diagnosis.

12. Page 8, Is the statement “Departments of Internal Medicine I and Gynecology at..” correctly mentioned in the method section of the abstract.

13. Page 8: The sentence needs to be ended after the "…detection of liver metastases (p<0.001)”. Please add a dot in the result section of the abstract or connect the following sentence properly.
