# Supplementary material for: Liver function indicators in patients with breast cancer before and after detection of hepatic metastases-a retrospective study
Source: PLoS One. 2023 Mar 3;18(3):e0278454. doi: 10.1371/journal.pone.0278454 (PMC9983906; doi:10.1371/journal.pone.0278454)
Supplement: Attachment — Submitted filename: Revision comments PLOS One Leser et al.docx [file pone.0278454.s004.docx]

**Comments**

Overall, the manuscript looks good. Authors have revised the manuscript and addressed the comments and concerns satisfactorily. The revised manuscript can be considered for publication in the journal PLOS One. However, few more corrections need to be made to further improve the manuscript:

1. Each enzyme or protein plot can be relabeled in alfa-numeric format in the Figure 2 and accordingly it can be mentioned in the figure legend and result section. Authors are suggested to label each graph from 2A-2G.

2. Legend and result have been combined for figure 2, which must be separated (Line 162-179). The description given in the revised manuscript is fitting well as result. Authors are advised to write figure legend after labeling each graph, as suggested in comment 1. Moreover, Authors must consider revising the legends of each figure, as the purpose of legend is to describe the figure, its axes, dimensions, and statistics, not to explain the results. Please consider revising the legends following legend template given below for figure 2:

Figure 2: Mean liver function protein level changes over time. The concentrations (Y-axis) of each enzyme, including AST (A), ALT (B), GGT (C), LDH (D), AP (E), and proteins, including Albumin (F), and Bilirubin (G), are plotted as bar graph, showing values 6-month prior, at the time of diagnosis, and 12-month post-diagnosis, represented on X-axis. Statistical analysis was performed using………..software and the p-values are represented as…………….
