# Supplementary material for: Liver function indicators in patients with breast cancer before and after detection of hepatic metastases-a retrospective study
Source: PLoS One. 2023 Mar 3;18(3):e0278454. doi: 10.1371/journal.pone.0278454 (PMC9983906; doi:10.1371/journal.pone.0278454)
Supplement: Attachment — Submitted filename: Review Leber 2.docx [file pone.0278454.s005.docx]

Reviewer #2: Overall, the manuscript looks good. Authors have revised the manuscript and addressed the comments and concerns satisfactorily. The revised manuscript can be considered for publication in the journal PLOS One. However, few more corrections need to be made to further improve the manuscript:

1. Each enzyme or protein plot can be relabeled in alfa-numeric format in the Figure 2 and accordingly it can be mentioned in the figure legend and result section. Authors are suggested to label each graph from 2A-2G. changed

2. Legend and result have been combined for figure 2, which must be separated (Line 162-179). The description given in the revised manuscript is fitting well as result. Authors are advised to write figure legend after labeling each graph, as suggested in comment 1. Moreover, Authors must consider revising the legends of each figure, as the purpose of legend is to describe the figure, its axes, dimensions, and statistics, not to explain the results. Please consider revising the legends following legend template given below for figure 2:

Figure 2: Mean liver function protein level changes over time. The concentrations (Y-axis) of each enzyme, including AST (A), ALT (B), GGT (C), LDH (D), AP (E), and proteins, including Albumin (F), and Bilirubin (G), are plotted as bar graph, showing values 6-month prior, at the time of diagnosis, and 12-month post-diagnosis, represented on X-axis. Statistical analysis was performed using………..software and the p-values are represented as……………. changed

Reviewer #3: This is a revision manuscript entitled “Liver function indicators in patients with breast cancer before and after detection of hepatic metastases” led by Carmen et al., and have significantly revised based on previous concerns raised by the reviewers. The manuscript is highly relevant topic given the poor clinical course of liver metastasis with breast cancer patients.
Minor comments to improve the manuscript.
a. Authors can consider revising their title to reflect that this is a retrospective changed

b. Also, BCLM and increased GGT and AP show that there increased biliary injury can be added in the manuscript. Further biliary injury has correlated with metastatic lesions has been observed in the manuscript added

c. Table 2 is confusing; number of samples can be in a separate column. Also, a dotted plot would with individual data would be a great addition to enhance the clarity of the data presented – At the beginning table 2 looked totally different. Reviewer 1 and 2 asked for this changes and they are happy with that except for figure legends and the need for alfa-numeric format.

d. Authors can add the details of obesity or NAFLD as a confounding variable in their analysis, if they have the data otherwise mentioning them in the limitations is fine – The other reviewers mentioned that point, so I`m sorry but I don´t have the datas and just mention it in the limitations.

e. Consider adding a subtitle of limitations before conclusion. added

f. Figure 2 legends needs the details of sample number and statistical analysis changed
